# Bimekizumab for recalcitrant pyoderma gangrenosum

**DOI:** 10.1016/j.jdcr.2025.12.018

**Published:** 2026-01-30

**Authors:** Christina Tolete, Adam Friedman, Joaquin Calderon, Leonardo Tjahjono

**Affiliations:** Department of Dermatology, George Washington University, School of Medicine and Health Sciences, Washington, District of Columbia

**Keywords:** bimekizumab, interleukin-17, neutrophilic dermatosis, pyoderma gangrenosum

## Introduction

Pyoderma gangrenosum (PG) is an inflammatory, ulcerative, and debilitating neutrophilic dermatosis for which there are no U.S. Food and Drug Administration-approved medications. Historical treatments include topical, intralesional, and systemic corticosteroids, cyclosporine, tumor necrosis factor, and interleukin (IL)-1 inhibitors.^1^ However, these treatments have limited efficacy and considerable side effects.

We present 2 cases of recalcitrant PG successfully treated with bimekizumab, an IL-17A/F inhibitor.

## Report of cases

**Fig 1:**
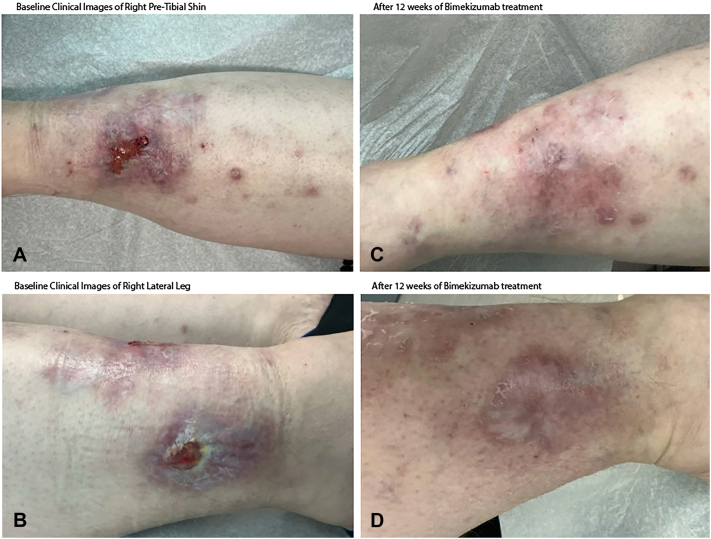
Clinical improvement of patient 1 with bimekizumab therapy. **A, B,** Baseline clinical images of the right pretibial shin and lateral leg demonstrate violaceous, undermined ulcerations with erythematous borders and fibrinous exudate consistent with pyoderma gangrenosum. **C, D,** After 12 weeks of bimekizumab treatment (320 mg every 2 weeks), there is marked improvement with re-epithelialization, decreased erythema, and resolution of ulceration.

A 42-year-old female with a history of severe hidradenitis suppurativa (HS) presented to our clinic with progressive right lower extremity pain and ulcerations of 7 months’ duration. Physical examination revealed painful ulcers with violaceous, underminable borders on the right pretibial shin and lateral leg (Fig 1, *A* and *B*). Punch biopsy from the border showed dense neutrophilic infiltrates with epidermal ulceration. Comprehensive evaluation – including stains, cultures, direct immunofluorescence, labs and malignancy screening, including colonoscopy – was negative for infection, vasculitis, autoimmune disease, Crohn’s disease, or malignancy. A diagnosis of idiopathic PG was made.

Prednisone 60 mg daily was initiated, along with a combination of either dapsone or adalimumab or mycophenolate mofetil or methotrexate. No combination was effective in allowing prednisone tapering below 40 mg, nor did they improve her pain level. Bimekizumab 320 mg every 2 weeks was therefore initiated given the persistent recalcitrance, and by week 6, pain improved, and by week 12, the patient reported negligible pain with marked improvement and healing of the PG and her severe HS (Fig 1, *C* and *D*). Prednisone was tapered and discontinued without recurrence of PG. Bimekizumab dosing was tapered to 320 mg every 4 weeks. At the 6-month follow up, resolution was sustained without reported adverse events while the bimekizumab regimen was maintained.

**Fig 2:**
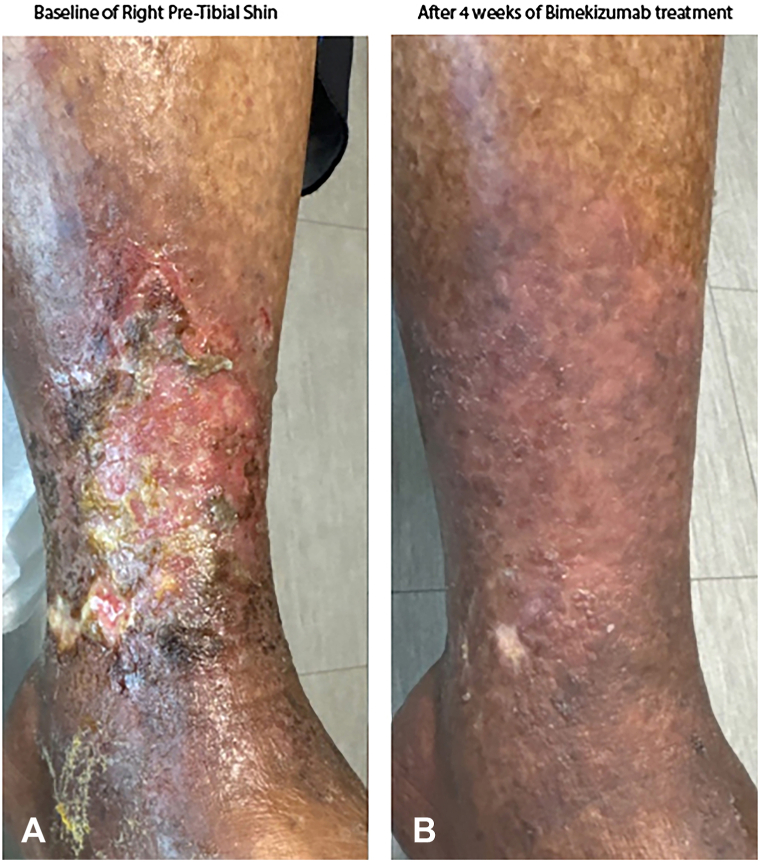
Clinical improvement of patient 2 with bimekizumab therapy. **A,** Baseline image of the right pretibial shin shows an extensive ulcer with crusting, exudate, and violaceous borders. **B,** After 4 weeks of bimekizumab treatment (320 mg every 2 weeks), there is near-complete re-epithelialization and resolution of inflammation and pain.

A 64-year-old female with a history of hypertension and hypercholesterolemia presented with an 18-month history of PG. An analogous work-up as per above was performed and punch biopsy results were again consistent with PG (Fig 2, *A*). Additionally, computed tomography angiography prior to presentation to our clinic did not show arterial insufficiency. The patient failed high dose prednisone, cyclosporine, adalimumab, and anakinra, each utilized for 4 months, with no improvement or ability to taper prednisone. Bimekizumab 320 mg every 2 weeks was initiated, and by week 8, pain improved, and at 4 months, complete resolution was achieved with successful complete tapering of both prednisone and bimekizumab to every 4-weeks (Fig 2, *B*). No adverse events were reported.

## Discussion

PG is a chronic inflammatory condition characterized by painful, debilitating inflammatory ulcers. It is commonly associated with systemic disorders, including hematologic dyscrasia, malignancy, infection, and inflammatory bowel disease. However, approximately 50% of cases, like in our patients, are idiopathic. PG is notoriously difficult to treat and significantly impairs quality of life. Dysregulation involving T-helper 17 cells, regulatory T cells, and IL-17 has been implicated in its pathogenesis.^2^ Broad and targeted IL inhibition, has been tried in small studies and case series with variable success, though paradoxical PG after treatment with IL-17 inhibitors has also been reported.^3^

Bimekizumab, IL-17A/F inhibitor, recently U.S. Food and Drug Administration-approved for the treatment of psoriasis and HS, may help restore T-helper 17/IL-17 axis imbalances and reduce neutrophilic infiltration, explaining its potential efficacy in PG.^2^ Ixekizumab has likewise shown benefit in case reports.^4^ In HS, dual IL-17A/IL17-F inhibition reduces neutrophil-related gene expression and migration more effectively than blocking either cytokine alone, making bimekizumab a strong option. In contrast, because IL-1ß mainly drives early neutrophil recruitment and activation, anakinra may be most useful only at disease onset.^5^ Importantly, several dermatologic biologics targeting tumor necrosis factor-α, IL-17, and IL-23 pathways have been associated with both clinical improvement in PG and paradoxical PG induction (Table I), underscoring the complexity of cytokine signaling in disease pathogenesis.

**Table I:** Summary of biologic agents used in dermatologic and inflammatory diseases that have been reported to either improve pyoderma gangrenosum or, paradoxically, trigger PG

Class	Biologics	Efficacy in PG	Causes paradoxical PG
TNF-i	Adalimumab	Yes^6^	Yes^7^
TNF-i	Infliximab	Yes^8^	Yes^9^
TNF-i	Etanercept	Yes^10^	Yes^11^
IL-17i	Ixekizumab	Yes^12^	Yes^13^
IL-17i	Brodalumab	Yes^14^	Yes^15^
IL-17i	Secukinumab	Yes^16^	Yes^17^

Reports include TNF-α inhibitors (adalimumab, infliximab, and etanercept) and IL-17 inhibitors (ixekizumab, brodalumab, and secukinumab).

*IL,* Interleukin*; PG,* pyoderma gangrenosum*; TNF,* tumor necrosis factor.

These cases highlight bimekizumab as a potential option for refractory PG. However, conclusions are limited by the nature of case reports and controlled studies are needed to evaluate bimekizumab as a treatment option for PG.

## Conflicts of interest

Dr Tjahjono has served as a consultant and/or speaker for Arcutis, Bristol Myers Squibb, Eli Lilly, Incyte, Leo pharma, and Galderma. Dr Friedman has served as a consultant or on the advisory board of La Roche Posay, Galderma, Kenvue, Microcures, Leo Pharma, Pfizer, Hoth Therapeutics, Zylo Therapeutics, Mino Labs, J&J, Arcutis, Lilly, UCB, Novartis, UCB, Regeneron/Sanofi, Takeda; they have also served as a speaker for Regeneron/Sanofi, J&J, Incyte, UCB, Galderma, Arcutis, Lilly, Pfizer, Novartis; they have also received grants from Pfizer, Lilly, Galderma, Incyte, J&J, Abbvie, Loreal, and Regeneron/Sanofi. Dr Calderon and Author Tolete have no conflicts of interest to declare.
